# Two new species in the subfamily Perlinae (Plecoptera, Perlidae) from China

**DOI:** 10.3897/zookeys.313.5460

**Published:** 2013-07-02

**Authors:** Hong–Liang Wang, Guo–Quan Wang, Wei–Hai Li

**Affiliations:** 1 Department of Plant Protection, Henan Institute of Science and Technology, Xinxiang, Henan 453003, China; 2 Department of Plant Protection, Guangxi University, Nanning, Guangxi 530004, China

**Keywords:** Plecoptera, Perlidae, *Neoperla*, *Kamimuria*, new species, China

## Abstract

Two species in the genera *Neoperla* and *Kamimuria* (Plecoptera: Perlidae) from China are described as new: *Kamimuria guangxia* **sp. n.**, and *Neoperla mesostyla* **sp. n.** The new species are compared to similar taxa.

## Introduction

*Kamimuria* and *Neoperla* (Plecoptera: Perlidae) are the most speciose genera within the subfamily Perlinae within China (Du et al. 1999, DeWalt et al. 2013). *Neoperla* is represented by more than 70 known species in China, comprising about 30% of species in the genus (DeWalt et al. 2013). These were described by Chu (1929), Du (1999, 2000a, b), Du and Sivec (2004, 2005), Du and Wang (2005, 2007), Du et al. (2001), Sivec and Zwick (1987), Wu (1935, 1938, 1948, 1962, 1973), Wu and Claassen (1934), Yang and Yang (1990, 1991), Yang and Yang (1992, 1993, 1995a, b, 1996, 1998), Li et al. (2011), Li and Wang (2011), Li et al. (2012), Li et al. (2012), Li and Li (2013) and Li et al. (2013). *Kamimuria* is represented by nearly 50 species in China, comprising about 70% of the described species within the genus (DeWalt et al. 2013; Sun and Du 2012; Li et al. 2012). In the present paper, we describe two additional Perlinae species as new to science: *Kamimuria guangxia* sp. n., and *Neoperla mesostyla* sp. n. from the Guangxi autonomous region. These species seem most closely related to congeners known from other southeastern Asian areas, as noted in the text. All types, including paratypes, are deposited in the Entomological Museum of China Agricultural University (CAU). Aedeagi were everted using the cold maceration technique of Zwick (1983) or Sivec et al. (1988).

## Taxonomy

### Kamimuria guangxia

Li & Wang sp. n.

urn:lsid:zoobank.org:act:5BF4961D-1520-418F-B086-F6F168097A51

http://species-id.net/wiki/Kamimuria_guangxia

Figs 1 – 3

#### Type material.

Holotype: male, originally labeled as China: Guangxi autonomous region, Tian’e County, Buliuhe River, light trap, 25.0005N, 107.1738E, 16 Aug. 2002, Ding Yang. Paratypes: 2 males, same data as holotype.

#### Male.

**Figure 1.**
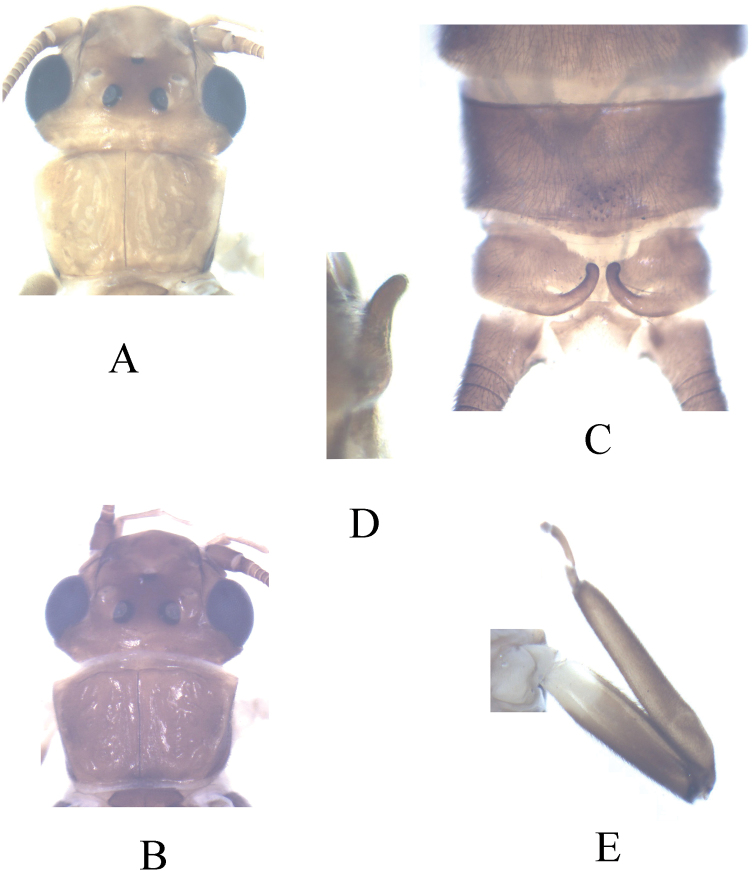
*Kamimuria guangxia* Li & Wang, sp. n. (male). **A** Head and pronotum, dorsal view (teneral specimen) **B** Head and pronotum, dorsal view (older specimen) **C** Terminalia, dorsal view **D** Hemitergal process, lateral view **E** Foreleg, lateral view.

Forewing length 14.8 – 15.0 mm. General body color dark brown. Head slightly wider than pronotum, generally brown with darker, quadrate interocellar region, the anterior corners of which extend laterally in teneral specimen, M-line pale (Figs 1A-B); compound eyes dark; antennae dark brown. Pronotum dark brown with rugose surface (Fig. 1A); wing membrane brown, veins darker; femora pale basally, otherwise dark (Fig. 1E).

#### Terminalia.

**Figure 2.**
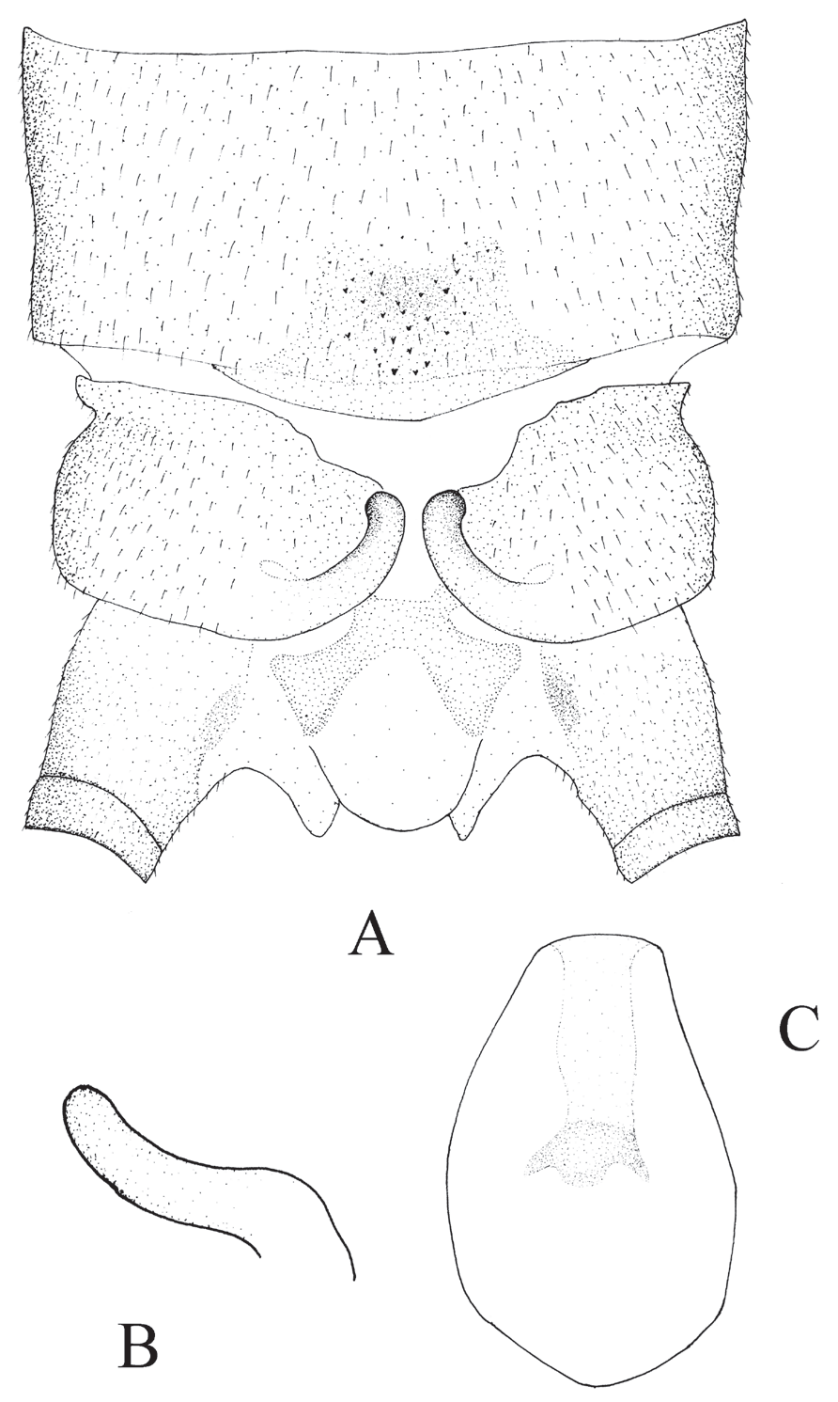
*Kamimuria guangxia* Li & Wang, sp. n. (male). **A** Terminalia, dorsal view **B** Hemitergal process, lateral view **C** Aedeagus before eversion, ventral view.

**Figure 3.**
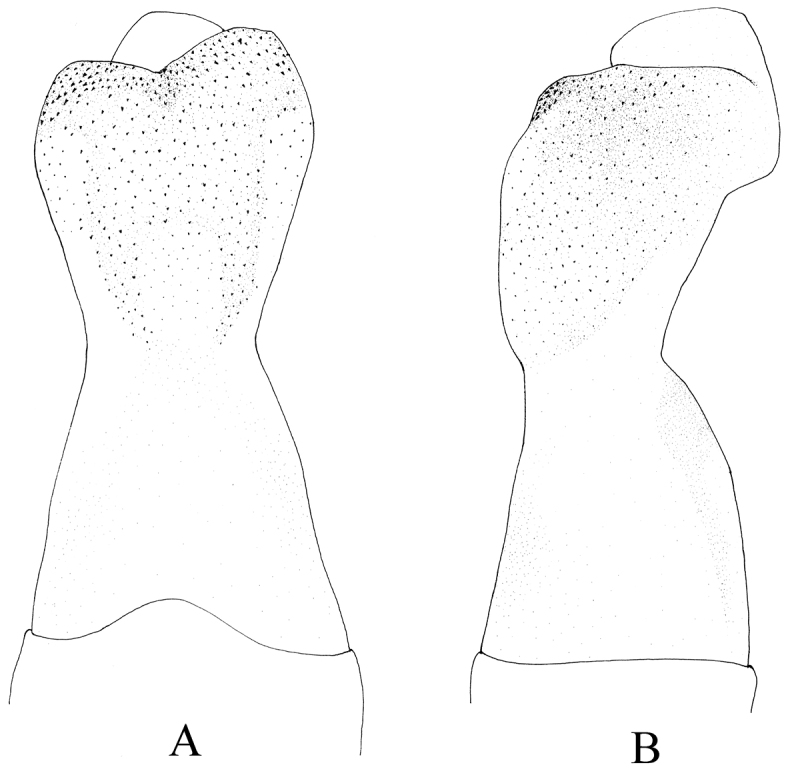
*Kamimuria guangxia* Li & Wang, sp. n. (male). **A** Aedeagus, dorsal view **B** Aedeagus, lateral view.

Hemiterga slender, finger-like, and slightly swollen apically, without hidden groove (Figs 2A–B). Tergum 9 with posteromedial patch of sensilla basiconical on a somewhater darker sclerite. Tergum 8 without sensilla patch. (Figs 1C, 2A). Setal brushes present on sterna 4–6. Aedeagus before eversion oval, apex tapering, hidden sac with apex darker and palm-like, with two lateral sharp claws and a median pad (Fig. 2C). Aedeagal sac membranous, medially constricted, apex expanded, heart-shaped and mostly covered with fine spinules, the apex and basal half of the sac bare (Fig. 3).

#### Female.

Unknown.

#### Etymology.

The specific epithet refers to Guangxi autonomous region where the type specimen was collected.

#### Distribution.

China (Guangxi).

#### Diagnosis.

The male of *Kamimuria guangxia* is characterized by the hemiterga being slightly swollen apically. The aedeagal sac is membranous, medially constricted, apex expanded, heart-shaped and mostly covered with fine to tiny spines (Figs 1C, 2B). The new species is similar to *Kamimuria atra* Sivec & Stark, 2008, a species known both from Vietnam and Thailand, in general body color and features of terminalia, but tergum 8 of the new species has no sensilla basiconica patch.

### Neoperla mesostyla

Li & Wang sp. n.

urn:lsid:zoobank.org:act:151A4084-8B37-424B-BD9A-8F8CB4A5857A

http://species-id.net/wiki/Neoperla_mesostyla

Figs 4 –5

#### Type material.

Holotype: male, China: Guangxi autonomous region, Mt. Jiuwanshan, Jiuren Station next to Rongjiang River, 950–1150 m, 25.0673N, 109.2563E, light trap, 3 Aug. 2003, Zhang Li-Li. Paratype: 1 male, same data as holotype.

#### Male.

**Figure 4.**
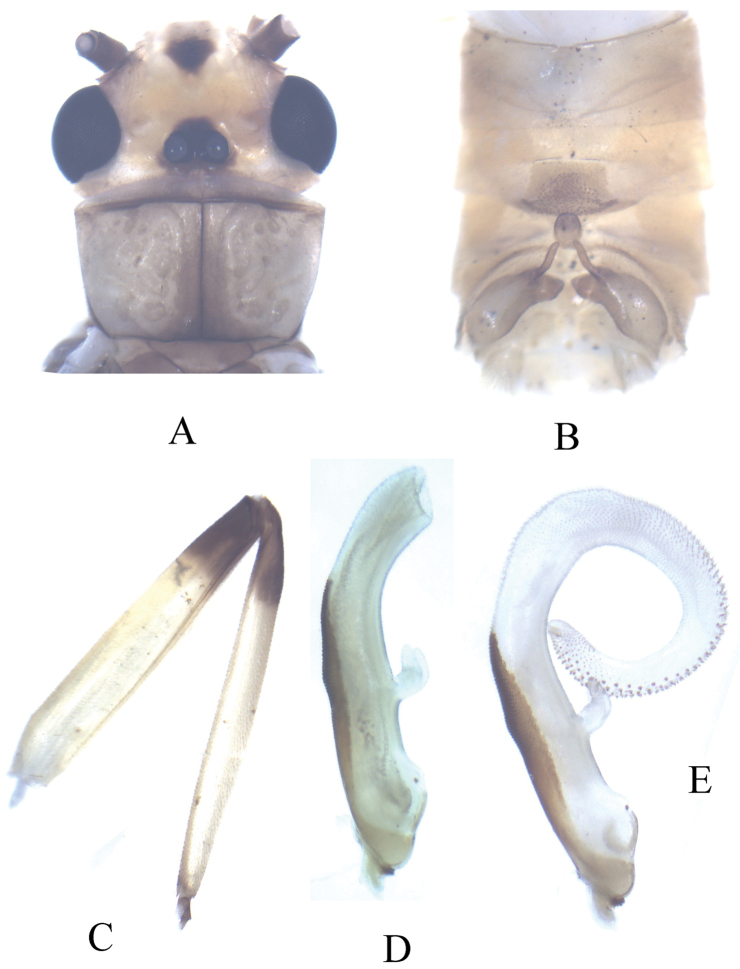
*Neoperla mesostyla* Li & Wang, sp. n. (male). **A** Head and pronotum, dorsal view **B** Terminalia, dorsal view **C** Hindleg (part of tarsi in this leg missing), lateral view **D** Aedeagus before eversion, lateral view **E** Aedeagus, lateral view.

Forewing length ca. 14.8 mm. General body color brown. Distance between ocelli nearly as wide as diameter of ocellus. Head slightly wider than pronotum, with a small triangular interocellar patch and another black triangular patch on frons (Fig. 4A); compound eyes dark; antennae dark brown. Pronotum pale brown with darker anterior and median stripes (Fig. 4A); wing membrane pale brown, veins dark; legs yellowish brown, distal fourth of femora, basal fifth of tibiae and tarsi darker (Fig. 4C).

#### Terminalia.

**Figure 5.**
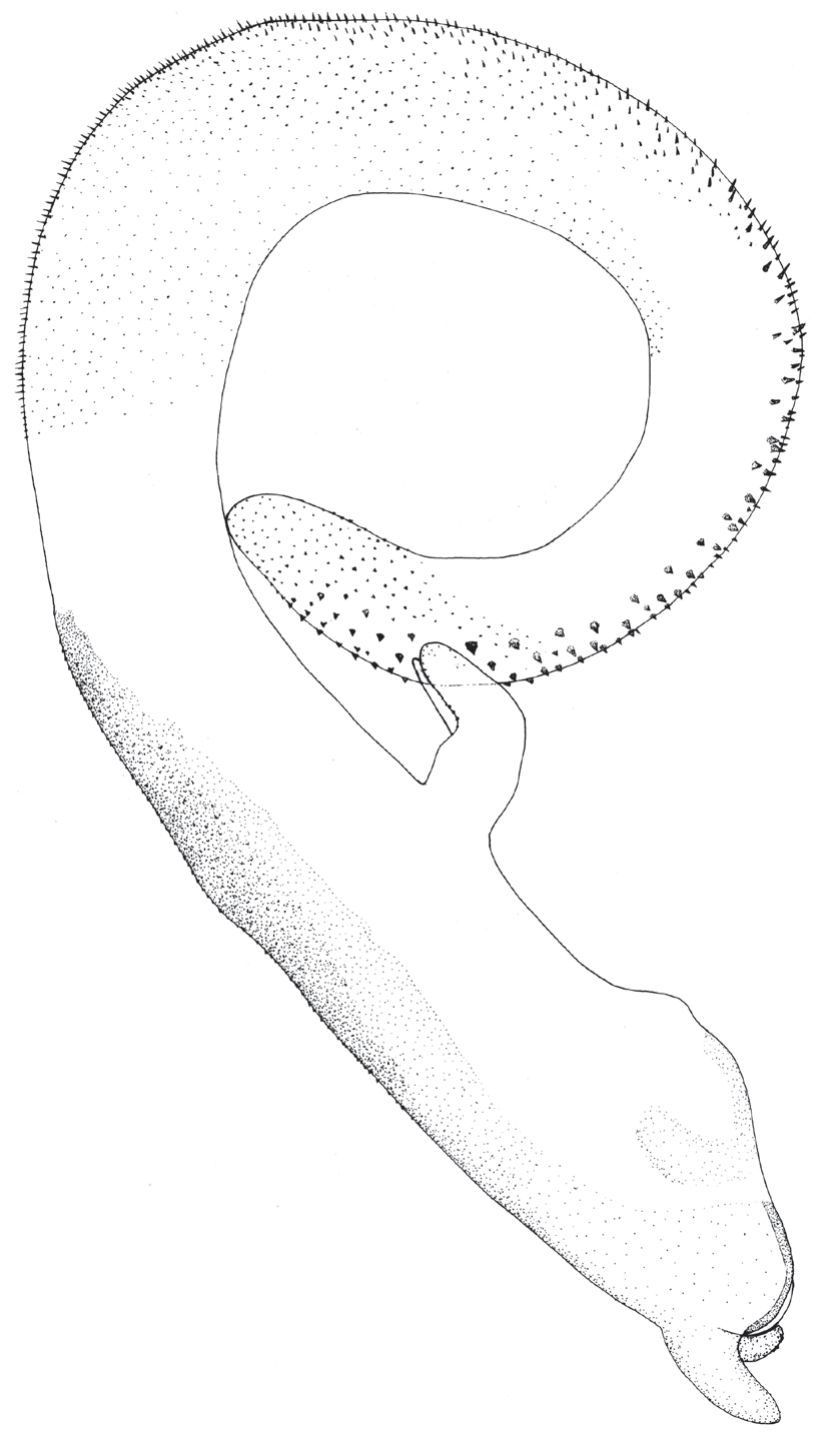
*Neoperla mesostyla* Li & Wang, sp. n. Male aedeagus, lateral view.

Process of tergum 7 forming a large subquadrate plateau, mostly covered with dense sensilla basiconica patches but with few sensilla basiconica at margins (Fig. 4B). Tergum 8 with an upcurved tongue-shaped process, with sparse ventral tiny spines. Tergum 9 without sensilla basiconica patches. Hemitergal lobes slender and curved laterally near midlength (Fig. 4B). Aedeagal tube plump (length 3× width at basal bulb), ventrally with a mesal bifurcate lobe bearing a basal common stem, dorsal surface heavily sclerotized, the pigmentation slightly expanded mesolaterally, membranous sac 1.5× as long as tube and gradually curved ventrad to form a loop; sharp to stout small spines occur along dorsal surface toward apex (Figs 4D, E, 5).

Zwick & Sivec defined the bulb side of the tube as being ventral, however, the sac of the new species forms a loop so that the spines of apical half of the dorsal surface are actually ventrally directed; we herein discuss dorsal or ventral surfaces of the sac as if it were straight, as such in *Neoperla flagellata* Li & Murányi (Li et al. 2012) and *Neoperla monacha* Stark & Sivec (Stark and Sivec 2008).

#### Female.

Unknown.

#### Etymology.

The specific epithet refers to the mesal position of the bifurcate lobe on the ventral surface of the aedeagal tube.

#### Distribution.

China (Guangxi).

#### Diagnosis.

The new species appears to belong to a well differentiated subgroup of the montivaga species group (Zwick 1983) that has as tube characteristics a dark, elongate sclerite dorsally and a bilobed, mostly membranous process ventrally (Zwick and Sivec 1985). Based on these features, several other species could also be assigned to this *diehli* subgroup: *Neoperla han* Stark, *Neoperla mnong* Stark, *Neoperla furcostyla* Li & Qin, *Neoperla forcipata* Yang & Yang and *Neoperla yao* Stark.

The new species is characterized by the aedeagal tube being short (≤3× width of bulb) and the ventral, bifurcate process being midlength on the tube. Additionally, the apical half of the sac is clothed with heavy spinules on the dorsal surface. *Neoperla diehli* has an elongate slender aedeagal tube (length ≥4× tube width), the ventral processes originate at the apex of the tube, and the heavy spinules are restricted to the tip of the sac (see Fig. 21 in Zwick and Sivec 1985). *Neoperla mesostyla* is easily distinguishable from other members of the group by its very long sac and relatively short Y-lobe. *Neoperla han* Stark and *Neoperla yao* Stark have elongate Y-lobes (Figs 6, 10 in Stark 1987); *Neoperla mnong* Stark, *Neoperla furcostyla* Li & Qin, and *Neoperla forcipata* Yang & Yang have a short or very short aedeagal sac (Fig. 7 in Stark 1987; Figs 2B, C in Li et al. 2013).

## Supplementary Material

XML Treatment for Kamimuria guangxia

XML Treatment for Neoperla mesostyla
